# A Computational Model of the Development of Separate Representations of Facial Identity and Expression in the Primate Visual System

**DOI:** 10.1371/journal.pone.0025616

**Published:** 2011-10-06

**Authors:** James Matthew Tromans, Mitchell Harris, Simon Maitland Stringer

**Affiliations:** Department of Experimental Psychology, University of Oxford, Oxford, Oxfordshire, United Kingdom; National Institute of Mental Health, United States of America

## Abstract

Experimental studies have provided evidence that the visual processing areas of the primate brain represent facial identity and facial expression within different subpopulations of neurons. For example, in non-human primates there is evidence that cells within the inferior temporal gyrus (TE) respond primarily to facial identity, while cells within the superior temporal sulcus (STS) respond to facial expression. More recently, it has been found that the orbitofrontal cortex (OFC) of non-human primates contains some cells that respond exclusively to changes in facial identity, while other cells respond exclusively to facial expression. How might the primate visual system develop physically separate representations of facial identity and expression given that the visual system is always exposed to simultaneous combinations of facial identity and expression during learning? In this paper, a biologically plausible neural network model, VisNet, of the ventral visual pathway is trained on a set of carefully-designed cartoon faces with different identities and expressions. The VisNet model architecture is composed of a hierarchical series of four Self-Organising Maps (SOMs), with associative learning in the feedforward synaptic connections between successive layers. During learning, the network develops separate clusters of cells that respond exclusively to either facial identity or facial expression. We interpret the performance of the network in terms of the learning properties of SOMs, which are able to exploit the statistical indendependence between facial identity and expression.

## Introduction

Single unit recording studies in non-human primates have revealed that a number of the visual processing areas of the brain appear to encode facial identity and facial expression across separate subpopulations of neurons. For example, it has been shown that the inferior temporal gyrus (TE) contained cells that were primarily responsive to facial identity, the adjacent superior temporal sulcus (STS) contained cells that primarily responded to facial expression, and the cells on the lip of the sulcus (TEm) tended to respond to expression and identity [1]. Cells responsive to facial identity are primarily found in inferior temporal cortex, while cells that respond to dynamic facial features such as facial expression are found in STS [2]. Orbitofrontal cortex (OFC) of non-human primates contains some cells that respond exclusively to changes in facial identity, while other cells respond exclusively to facial expression [3]. Similar cells have been found in the amygdala of non-human primates, which respond to either facial identity or facial expression [4].

Further evidence of physically separate visual representations of facial identity and expression comes from fMRI adaptation (fMRIa) studies in humans. Using fMRIa, functional dissociations within the STS have been demonstrated [5]. Specifically, cells in lateral right fusiform cortex and pSTS were released from adaptation upon changes to facial identity, while cells in more anterior STS were released from adaptation upon changes to facial expression. These findings are consistent with other neuroimaging studies, including [6]–[8].

How might the primate visual system develop physically separate representations of facial identity and expression given that the visual system is always exposed to simultaneous combinations of facial identity and expression during learning? Previous research has shown that Principal Component Analysis (PCA) can extract and categorise facial cues related to facial identity and expression [9], [10]-for a review see [11]. However, these computational methods are not based on biologically plausible models of brain function. In this paper, we show for the first time how separate visual representations of facial identity and expression could develop in a biologically plausible neural network architecture using associative Hebbian learning.

In the simulations described below, images of faces with different identities and expressions are shown to a neural network model, VisNet, of the ventral visual pathway [12]–[17]. The VisNet model has a biologically plausible neural network architecture. The version of the VisNet architecture used in this paper consists of a feedforward series of four Self-Organising Maps (SOMs). During learning, the feedforward synaptic weights are updated by associative Hebbian learning. A key aspect of the model for biological plausibility is that learning is unsupervised, that is, we do not explicitly tell the network the identity or expression of the current face during training.

In this present study, the network is trained with complete cartoon faces, which convey information about both facial identity and expression simultaneously. The face images are comprised of two types of continuously varying facial features. The eyes and nose convey where the face lies within a uni-dimensional continuum of identities, while the mouth and eyebrows convey where the face lies within a uni-dimensional continuum of expressions. After training, the output layer of the network has developed separate clusters of cells that respond exclusively to either facial identity or facial expression. Individual neurons that learn to encode identity fire selectively to a small region of the space of identities regardless of expression. Similarly, individual neurons that learn to represent expression fire selectively to a small region of the space of expressions regardless of identity. We interpret the performance of the network in terms of the learning properties of SOMs.

### How might the primate brain form separate visual representations of facial identity and expression?

Recent research has shown how VisNet can exploit the statistics of natural visual input in order to learn separate visual representations of objects despite the fact that multiple objects were always presented together during training [16], [17]. During presentation, the features that make up an object occur together more frequently compared to the features that make up different objects. The frequency with which the objects are presented together during training denotes the level of correlation between features from different objects. However, the features that comprise individual objects are always seen together and so are more highly correlated. In this situation, a competitive network will form representations of individual objects, rather than the combinations of objects seen during training. The same effects should also be present in SOMs.

**Figure 1:**
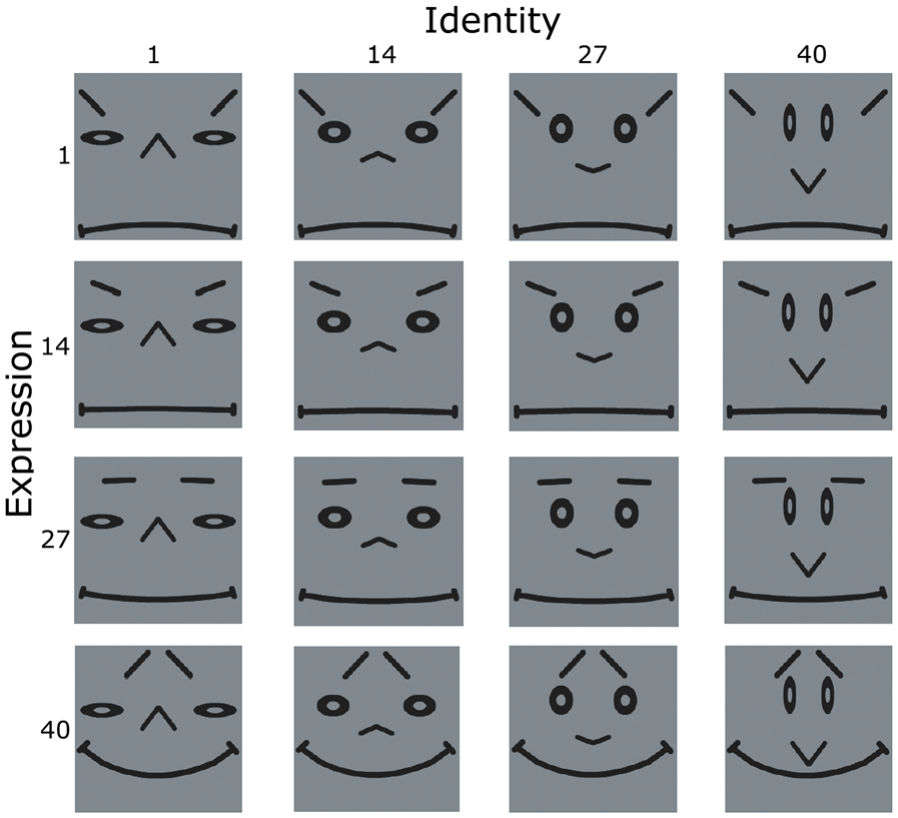
Cartoon face stimuli. A selection of 16 cartoon face stimuli presented to VisNet where identity (represented by the shape and location of the eyes and nose) changes along the horizontal dimension (left to right) and expression (represented by the shape and location on the mouth and eyebrows) changes along the vertical dimension (top to bottom). A total of 1600 faces were produced by combining all possible combinations of the 40 identities and 40 expressions.

In this paper we show how similar mechanisms, which exploit the visual statistics of facial identity and expression, may cause VisNet to form separate representations of identity and expression during training with complete faces. In this new situation, facial identity and expression are each modelled as separate spaces rather than discrete objects. An example is shown in Fig. 1, which shows a selection of cartoon faces composed from particular combinations of 40 identities and 40 expressions. In this simplified test case, identity and expression are represented by different facial features. Specifically, identity is represented by the shape and location of the eyes and nose, while expression is represented by the mouth and eyebrows. The space of identities varies along the horizontal dimension, while the space of expressions varies along the vertical dimension. Each space can be varied independently of the other.

During training, all 1600 possible faces are presented to VisNet. During presentation, the features that make up a particular identity always occur together. The Hebbian learning rule will encourage output neurons to learn to respond to input features that tend to occur together simultaneously during training. However, the features that make up that identity are paired with each of the different expressions on different occasions. Therefore, the features that make up the identity occur together far more frequently than they occur with the features that make up any particular expression. This statistical decoupling between the particular identity and any of the expressions prevents an output neuron from learning to respond to a combination of the identity and any one of the expressions. In this situation, a SOM will form representations of individual identities, rather than the combinations of identity and expression seen during training.

Similarly, the features that make up a particular expression always occur together. Moreover, the features that make up that expression are paired with each of the different identities on different occasions. Therefore, the features that make up the expression occur together far more frequently than they occur with the features that make up any particular identity. This prevents an output neuron from learning to respond to a combination of the expression and any one of the identities. Therefore, a SOM will similarly form representations of individual expressions.

We investigate the effects of a self-organising map (SOM) by introducing short-range excitatory connections and long-range inhibitory connections [18], [19] to the original VisNet architecture. This lateral connectivity of the SOM uses a ‘Mexican-hat’ profile and encourages spatially proximal neurons to develop similar response properties due to their mutual excitation while neurons that are relatively far apart will experience mutual inhibition. This drives competition to create separate pools of functionally distinct neurons, which encode either the space of identities or the space of expressions.

In this first study, we have investigated how this process might operate with idealised cartoon faces, in which facial identity and expression are unidimensional spaces and represented by non-overlapping sets of visual features. However, we propose that, as long as there is sufficient statistical decoupling between facial identity and expression, then a similar mechanism will operate with more realistic face stimuli, in which the dimensions of identity and expression are more complex and the two spaces are represented by a common set of overlapping visual features.

The computer simulations presented in this paper are carried out using an established model, VisNet, of the ventral visual pathway. As discussed above, experimental studies in non-human primates have shown a dissociation between the representations of facial identity and expression across a number of visually-responsive brain areas both within and outside the ventral visual pathway. However, we propose that the learning mechanisms demonstrated in our model below, in which SOMs are able to exploit the statistical decoupling between facial identity and expression, may still operate across a more complex network of visually-responsive areas in the brain.

## Results

The VisNet model was trained on all possible combinations of expression and identity to create a total of 1600 faces. The results presented below show that the network was able to form separate localised clusters of neurons in the fourth (output) layer which represented the two independent visual spaces, identity and expression, even though they are presented together as complete faces during training. Crucially, neurons in the fourth layer of the network learned to respond to different portions of each visual space. Some neurons learned to respond to a small part of the space of identity irrespective of expression. Similarly, other neurons learned to respond to a small part of the space of expression irrespective of identity.

### Analysis of firing rate responses

**Figure 2:**
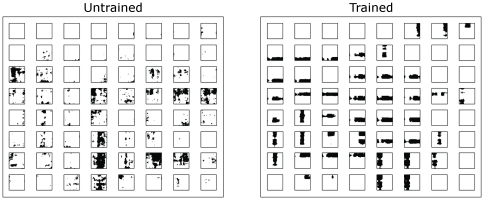
Neuronal firing rate responses to complete faces. The firing rate responses of an 8×8 sample of fourth (output) layer cells are shown before training (left) and after training (right) with the full set of 1600 complete faces. Each of the 8×8 sub-plots represents an individual cell in the output layer, and shows its firing-rate profile to all 1600 faces presented during testing. For each subplot, the horizontal axis denotes the position of the test face within the identity space and the vertical axis denotes the position within the expression space. The responses of the neuron are represented on a grey scale where black indicates high firing. After training, some individual neurons learned to respond to local portions of their preferred space, either identity or expression, irrespective of the position in the alternative space. This results in the appearance of vertical or horizontal ‘bars’ of activation in the subplots. If a neuron responds selectively to a local region of the identity space, then this results in a vertical bar in the subplot. In contrast, if a neuron responds to a local region within the expression space, then this results in a horizontal bar. Furthermore, due to the effects of the SOM architecture, cells with similar response profiles form close together. This causes spatial clustering of cells that respond preferentially to either facial identity or facial expression.

Fig. 2 shows neuronal firing rate responses to complete faces. The firing rate responses of an 8×8 subset of fourth (output) layer cells are shown before training (left) and after training (right) with the full set of 1600 complete faces. The 8×8 neurons are represented by 64 2D response plots, one per neuron. Each 2D response plot shows the neuron's firing-rate response to all 1600 possible faces. For each subplot, the x-axis is bound between 1 and 40, where each discrete point along the horizontal axis represents a different transform of the identity space. Similarly, each discrete point along the vertical axis represents a different transform of the expression space. The firing-rate of each neuron is then plotted for the different combinations of the 40 identities and 40 expressions. Strong firing is depicted by a dark shading.

In the untrained condition, cells responded fairly randomly due to the untrained random weight structure in the network. However, after training, some neurons responded to local portions of their preferred space, either identity or expression, irrespective of the position in the alternative space. This is evidenced by the appearance of vertical or horizontal ‘bars’ of activation in the subplots. If a neuron responds selectively to a local region of the identity space, then this results in a vertical bar in the subplot. Alternatively, if a neuron responds to a local region within the expression space, then this results in a horizontal bar. Furthermore, due to the effects of the SOM architecture, cells with similar response profiles form close together. This causes spatial clustering of cells that respond preferentially to either facial identity or facial expression. The firing rates tended to be rather binarised, i.e. near zero (white) or one (black), because of the steep slopes of the sigmoid transfer functions used in the present simulations.

**Figure 3:**
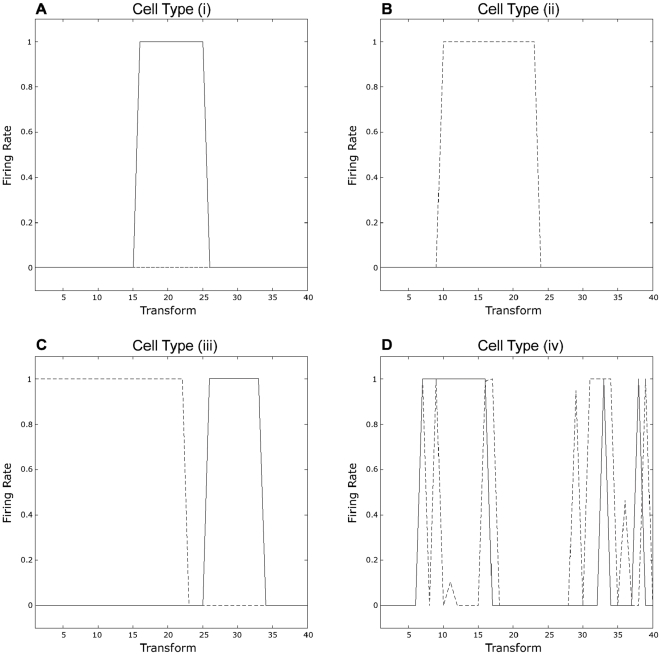
Neuronal firing rate responses to either identity or expression. First the network is trained with all 1600 complete faces. Then the network is tested with the visual features that represent either the 40 transforms of facial expression or the 40 transforms of facial identity presented separately. The figure displays the responses of four different kinds of typical output cells: (a) a cell that responds exclusively to a portion of the identity space; (b) a cell that responds exclusively to a portion of the expression space; (c) a cell that responds to both portions of the identity and expression space; and (d) a cell that responds to multiple portions of either or both spaces. Cell responses to the 40 transforms of identity are plotted with the solid line, and cell responses to the 40 transforms of expression are plotted with the dashed line. For each cell, the firing-rate (0–1) is plotted on the y-axis against the identity/expression number (1–40) on the x-axis.

We also tested the model with each of the 40 identities and 40 expressions used to generate the 1600 faces presented during training. Fig. 3 shows neuronal firing rate responses to either identity or expression. The network is first tested with each one of the 40 images that comprise the identity space, and then tested with the 40 images that comprise the expression space. The figure displays the responses of four different kinds of typical output cells: (a) a cell that responds exclusively to a portion of the identity space; (b) a cell that responds exclusively to a portion of the expression space; (c) a cell that responds to both portions of the identity and expression space; and (d) a cell that responds to multiple portions of either or both spaces. Cell responses to the 40 transforms of identity are plotted with the solid line, and cell responses to the 40 transforms of expression are plotted with the dashed line. For each cell, the firing-rate (0–1) is plotted on the y-axis against the identity/expression number (1–40) on the x-axis.

We also investigated whether cells that responded to a part of either the expression space or the identity space had learned to respond to a conjunction of the corresponding input features. By tracing the synaptic weights after training from cells in layer four back to the input filters, we found that this was indeed the case. For example, in the case of identity, we found that individual cells in layer four, which responded selectively to a particular part of the identity space, received strengthened connections from corresponding conjunctions of both the eyes and the nose. In the case of expression, we found that cells received strengthened connections from all parts of the mouth including the two corners of the mouth, although with weaker connections from the eyebrows.

### Information analysis

**Figure 4:**
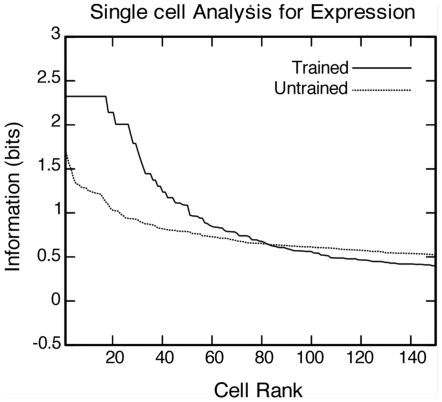
Information analysis for expression. Analysis of information about expression conveyed by fourth (output) layer cells before and after training on 1600 complete faces. The information analysis was performed as described in the Methods. The plot shows the amount of single cell information carried by individual output cells in rank order. In the trained condition, 17 neurons provided maximal information of 2.32 bits. In the untrained condition, no cells reached maximal information.

Fig. 4 shows the results of analysing the amount of information about expression conveyed by fourth (output) layer cells before and after training on 1600 complete faces. The information analysis was performed as described in the Methods. This analysis involved quantising the expression space into five separate contiguous blocks. The maximal amount of information possible in this case is 2.32 bits. Fig. 4 shows the amount of single cell information carried by individual output cells in rank order. In the trained condition, 17 neurons provided the maximal information of 2.32 bits. These 17 neurons responded selectively to a particular quantised portion of the expression space irrespective of the identity. In the untrained condition, no cells reached maximal information.

It can be seen that training the network on all 1600 complete faces has led to a dramatic increase in the information carried by the fourth layer neurons to the expression of the current face irrespective of identity.

**Figure 5:**
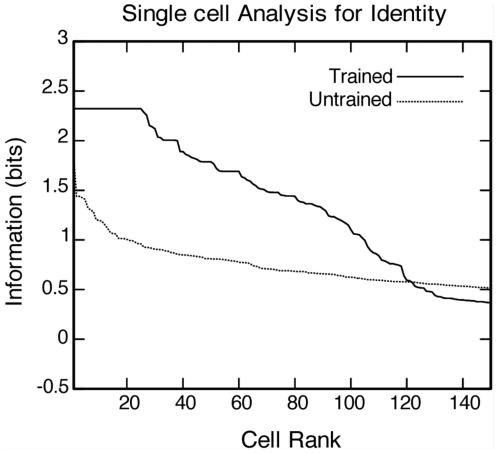
Information analysis for identity. Analysis of information about identity conveyed by fourth (output) layer cells before and after training on 1600 complete faces. The information analysis was performed as described in the Methods. The plot shows the amount of single cell information carried by individual output cells in rank order. In the trained condition, 25 neurons provided maximal information of 2.32 bits. In the untrained condition, no cells reached maximal information.

Fig. 5 shows the results of analysing the amount of information about identity conveyed by fourth (output) layer cells before and after training on 1600 complete faces. This analysis involved quantising the identity space into five separate contiguous blocks as described in the Methods. The plot shows the amount of single cell information carried by individual output cells in rank order. In the trained condition, 25 neurons provided the maximal information of 2.32 bits. In the untrained condition, no cells reached maximal information.

In summary, Figs. 4 and 5 confirm that, after training on the 1600 complete faces, the output layer cells have learned to convey large amounts of information about either facial identity or expression. This is because different subpopulations of cells in the output layer have learned to respond selectively to just one of the visual spaces, identity or expression, irrespective of the current transform of the other space. That is, some cells respond exclusively to a particular portion of the identity space, while other cells respond exclusively to a particular portion of the expression space.

## Discussion

A number of experimental studies have shown that physically separated subpopulations of neurons develop in the visually responsive areas of the primate brain that respond primarily either to facial identity or to facial expression [1]–[4]. In this paper we have provided a biologically plausible neural network model of how these separate clusters of neurons might develop through learning. The results of our simulations were confirmed by examination of the firing rate responses of the output cells, as well as analysis of the information carried by these cells about facial identity and expression. This is a non-trivial problem because the visual system is always exposed to combinations of facial identity and expression during learning. Our model solves the problem by exploiting how SOMs self-organise their output neuronal responses when they are trained on statistically independent visual input spaces such as facial identity and expression.

The learning principles demonstrated in this paper can also be applied to other problems in vision, such as learning to recognise different parts of the body [20], learning separate representations of two different objects that are always seen together but which move independently [21], or learning to classify gender.

Our simulations were performed using an established model, VisNet, of the ventral visual pathway. Experimental studies have demonstrated a dissociation between representations of facial identity and expression occurs across a number of visually responsive areas of the primate brain, both within and outside the ventral visual pathway. Nevertheless, our model has demonstrated biologically plausible learning mechanisms, specifically how learning in SOMs is shaped by the statistical independence between facial identity and expression, that may operate across these different but connected brain areas. In further simulation work we will explore how more architecturally detailed models of these interconnected areas of the brain develop their heterogeneous representations of facial identity and expression.

The results presented in this paper are robust. Altering the learning rates over three orders of magnitude did not qualitatively alter our findings. We also explored the effects of varying levels of global competition within the model by adjusting the sparseness percentile values. The model performed well with a sparseness of 4% to 10% within each layer, that is when 4% to 10% of the neurons were allowed to fire during any given stimulus presentation. Large variations in learning rate, sparseness, and the width of the SOM still produced spatially organised clusters of cells that were responsive to either facial identity or expression.

A spatial filter 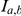 that simulated short-range excitatory and long-range inhibitory connections was introduced to the VisNet model to create the SOM network architecture. There is some anatomical evidence demonstrating that lateral inhibitory connections in V1 tend to project over a smaller proximity than excitatory connections [22]–[24]; this is the opposite of the SOM architecture implented in VisNet. However, if lateral inhibitory connections are rapid, a Mexican-hat style functional architecture may still be achieved [25]. The SOM architecture implemented in this paper is similar to others that are successful in replicating a range of experimental findings [26]–[28].

The tuning profiles of cells shown in Fig. 3 are consistent with non-human primate single cell recording data from [29]; in their study only 6.7% of cells responded to facial expression and identity. However, more recent research has shown that in the monkey amygdala, 64% of recorded cells responded to both facial expression and identity [4]. This cell type is presented in Fig. 3 (iii). Indeed, fMRIa studies with human subjects report clusters of cells in the fusiform face area (FFA) that respond to both identity and expression [8]. This type of heterogeneous cell type is consistent with our simulations, which produced four types of cell in the output layer.

A number of studies have revealed that neonates are able to mimic certain facial gestures [30], [31]. Furthermore, neonates show preference for familiar faces over novel faces, suggesting they can recognise different identities [32]. However, the question of innate functional segregation remains an open issue. The model we have presented in this paper shows how a functional segregation between facial identity and expression could be learned through visual experience without requiring any innate functional segregation.

The VisNet architecture used in this study has a number of limitations in terms of biological accuracy. For example, the current VisNet model lacks backprojections, which are a major feature of the ventral visual pathway in the primate brain. In future developments of the VisNet model we intend to explore the effects of these additional connections during learning. Also, because the current model is purely feedforward, it is possible to train the layers one at a time from layer 1 to 4. However, with the introduction of backprojections, all of the layers will need to be trained simultaneously. Moreover, the neurons in the current model are rate-coded with rather binarised firing rates. In future studies, we will implement integrate and fire neurons, which explicitly model the times of action potentials. This in turn will allow us to explore the effects of spike time dependent plasticity on visually-guided learning in the network.

In this paper we used carefully designed cartoon faces. This was to allow us to begin by exploring the simplified situation in which the visual spaces of facial identity and expression are independent, uni-dimensional, and non-overlapping on the retina. However, real faces are more complex in a number of ways. First, with real faces, expression may not be entirely independent of identity. Secondly, identity and expression are not simple uni-dimensional spaces. Thirdly, individual facial features such as the mouth may convey information about both identity and expression simultaneously. In this case, the visual representations of identity and expression are encoded by overlapping sets of retinal input neurons in a more distributed manner. Therefore, in future work, we intend to test how the learning mechanisms demonstrated in the simulations above might work with face images that are more realistic in all of these respects. For example, more realistic faces can be produced by the FaceGen modeller software package, which is used in a number of fMRI studies into the separation of identity and expression [8]. We will eventually test the model on real faces. This will allow us to assess the network's ability to generalise when tested with faces not presented during training. However, we expect that this will require VisNet's retina to be substantially increased in size in order to provide a sufficiently detailed input representation of the visual features encoding facial identity and expression.

## Methods

### The VisNet Model

The original VisNet architecture [33] is based on the following: (i) A series of hierarchical competitive networks with local graded inhibition. (ii) Convergent connections to each neuron from a topologically corresponding region of the preceding layer, leading to an increase in the receptive field size of neurons through the visual processing areas. (iii) Synaptic plasticity based on a Hebb-like associative learning rule.

In this paper, we implement a self-organising map (SOM) [18], [19] within each layer in place of the original competitive network architecture. That is, in our model simulations we adjusted the local graded inhibition within a layer to incorporate short-range excitation and longer-range inhibition.

**Figure 6:**
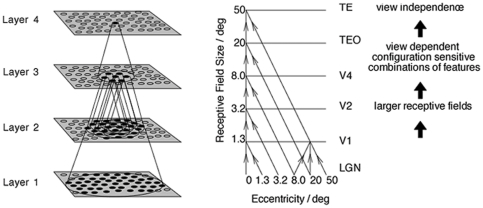
VisNet model. Left: Stylised image of the four layer VisNet network. The four layers of the network represent successive stages in the ventral visual pathway of the primate brain: V2, V4, TEO (posterior inferior temporal cortex) and TE (anterior inferior temporal cortex). The synaptic connections to V2, the first layer of the network, are derived from an array of input filters with the response characteristics of V1 simple cells. The V1 input filters are used to process the raw visual images to provide input to the first layer of the network. The layer of V2 cells then sends feedforward connections to the V4 layer. Similarly, V4 sends feedforward connections to TEO, and TEO sends feedforward connections to TE. During training with visual images, the feedforward synaptic connections into each of the four stages (V2, V4, TEO and TE) are modified by Hebbian learning. Convergence through the network is designed to provide fourth layer (TE) neurons with information from across the entire input retina. Right: Convergence of feedforward connections through successive stages of the ventral visual pathway. The receptive field size of neurons increases through successive layers. The final layer, TE, receives visual input from across the whole retina.

This VisNet model consists of a hierarchical series of four layers of SOMs, corresponding to V2, V4, the posterior inferior temporal cortex, and the anterior inferior temporal cortex, as shown in Fig. 6. The forward connections to individual cells are derived from a topologically corresponding region of the preceding layer, using a Gaussian distribution of connection probabilities. These distributions are defined by a radius which will contain approximately 67% of the connections from the preceding layer. The values used are given in Table 1.

**Table 1:** Network dimensions.

	Dimensions	Number of Connections	Radius
Layer 4	32×32	100	12
Layer 3	32×32	100	9
Layer 2	32×32	100	6
Layer 1	32×32	272	6
Retina	128×128×32	-	-

Network dimensions showing the number of connections per neuron and the radius in the preceding layer from which 67% are received.

Before the visual stimuli are presented to the network's input layer, they are pre-processed by a set of input filters which accord with the general tuning profiles of simple cells in V1. The filters provide a unique pattern of filter outputs for each visual face stimulus, which is passed through to the first layer of VisNet. The input filters used are computed by weighting the difference of two Gaussians by a third orthogonal Gaussian according to the following:
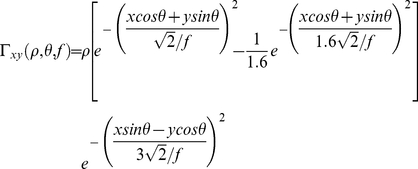
(1) where *f* is the filter spatial frequency, *θ* is the filter orientation, and *ρ* is the sign of the filter, i.e. ±1. Individual filters are tuned to spatial frequency (0.0625 to 0.5 cycles/pixel); orientation (0° to 135° in steps of 45°); and sign (±1). The number of layer 1 connections to each spatial frequency filter group is given in Table 2. Past neurophysiologcal research has shown that models based on difference-of-Gaussians functions can account for the variety of shapes of spatial contrast sensitivity functions observed in cortical cells better than those based on the Gabor function or the second differential of a Gaussian. They do have more free parameters, but unlike other models, they provide a detailed description of the organization of subregions of the receptive field that is consistent with the physiological constraints imposed by earlier stages in the visual pathway [34].

**Table 2:** Layer 1 connectivity.

Frequency	0.5	0.25	0.125	0.0625
Number of Connections	201	50	13	8

The numbers of connections from each spatial frequency set of filters are shown. The spatial frequency is in cycles per pixel.

The activation *h_i_* of each neuron *i* in the network is set equal to a linear sum of the inputs *y_j_* from afferent neurons *j* weighted by the synaptic weights *w_ij_*. That is,
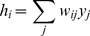
(2)where *y_j_* is the firing-rate of neuron *j*, and *w_ij_* is the strength of the synapse from neuron *j* to neuron *i*.

In this paper, we run simulations with a self-organising map (SOM) implemented within each layer. Short-range excitation and long-range inhibition are combined to form a ‘Mexican-hat’ spatial filter, which is constructed as a difference of two Gaussians as follows:
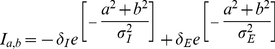
(3)


To implement the SOM, the activations *h_i_* of neurons within a layer are convolved with the spatial filter, 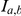, where 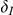 controls the inhibitory contrast and 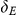 controls the excitatory contrast. The width of the inhibitory radius is controlled by 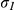 while the width of the excitatory radius is controlled by 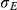. *a* and *b* index the distance away from the centre of the filter. The lateral inhibition and excitation parameters are given in Table 3.

**Table 3:** Lateral Connectivity Parameters.

Layer	1	2	3	4
Excitatory Radius, 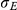	0.7	0.55	0.4	0.6
Excitatory Contrast, 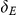	5.35	33.15	117.57	120.12
Inhibitory Radius, 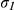	1.38	2.7	4.0	6.0
Inhibitory Contrast, 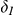	1.5	1.5	1.6	1.4

Lateral inhibition and excitation parameters for the SOM.

Next, contrast enhancement is applied by means of a sigmoid activation function
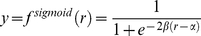
(4)where *r* is the activation (or firing-rate) after applying the SOM filter, *y* is the firing-rate after contrast enhancement, and *α* and *β* are the sigmoid threshold and slope respectively. The parameters *α* and *β* are constant within each layer, although *α* is adjusted to control the sparseness of the firing-rates. For example, to set the sparseness to, say, 5%, the threshold is set to the value of the 95th percentile point of the activations within the layer. The parameters for the sigmoid activation function are shown in Table 4. These are robust values found to operate well for this experiment.

**Table 4:** Sigmoid parameters.

Layer	1	2	3	4
Percentile	95	95	95	95
Slope *β*	190	40	75	26

The sigmoid parameters used to control the global inhibition within each layer of the model.

The inhibitory part of the SOM filter and contrast enhancement stages of the VisNet model aim to simulate the function of inhibitory interneurons. In the brain, inhibitory interneurons effect direct competition between excitatory cells within each layer of the ventral visual pathway. The way in which contrast enhancement is currently implemented in VisNet allows us to control the sparseness of firing-rates within each layer. This is a useful aspect of the model, which allows us to explore the effects of sparseness on network performance.

An important property of the model is that the learning at synaptic connections between cells is unsupervised. That is, during training, the expression or identity of the current face is not explicitly specified to the network to guide learning. The co-activation of neurons in two successive layers causes their synaptic connection to become strengthed, according to a Hebbian learning rule,
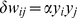
(5)where 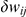 is the increment in the synaptic weight *w_ij_*, *y_i_* is the firing-rate of the post-synaptic neuron *i*, 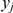 is the firing-rate of the pre-synaptic neuron *j*, and *α* is the learning rate. To restrict and limit the growth of each neuron's synaptic weight vector, *w_i_* for the *i* th neuron, its length is normalised at the end of each timestep during training as is usual in competitive learning [35]. Normalisation is required to ensure that the same set of neurons do not always win the competition. Neurophysiological evidence for synaptic weight normalization is provided by [36].

### Stimuli

To train the VisNet model we used computer-generated images of 2D faces, shown in Fig. 1. The use of carefully-constructed cartoon faces allowed us to investigate in the most controlled way the hypothesis outlined in the Introduction. In this initial study, we examine the simplest case, in which the identity and expression spaces are unidimensional and different facial features encode either identity or expression but not both. First we created 40 continuously varying transforms of identity by exclusively varying the shape and location of the nose and eyes (Fig. 1: left to right). Then we created 40 continuously varying transforms of expression by varying the shape and location of the mouth and eyebrows (Fig. 1: top to bottom). The different identities and expressions were then combined to create 40×40 = 1600 combinations of facial identity and expression. The final images were presented on a 128×128 pixel background each as a 256 grey level image.

### Training procedure

To train the network, all identity and expression combinations were presented creating a total of 1600 possible faces. One training epoch comprises all 1600 face presentations. At each presentation of a face to the network, the activation of individual neurons is calculated, then their firing-rate is calculated and finally their synaptic weights updated in accordance with the procedure outlined above.

To reduce the computational expense, we exploited the feedforward architecture of the model by training the network one layer at a time from layer 1 through 4. The feedforward architecture means that each layer must wait for learning in the previous layer to converge before it can do the same. Therefore, in previous studies, training the network one layer at a time has been found not to affect the qualitative performance of the network [37]. We used 50, 100, 100, 75 training epochs for layers 1, 2, 3 and 4 respectively. We explored the performance of the network using a SOM within each layer. In all experiments, the learning rate of the model was set to 0.1 and the sparseness was set to 0.05. Other values were explored (not presented) and receive comment in the Discussion.

### Testing procedure

After training all four layers of the network on the 1600 faces comprising unique combinations of identity and expression, the performance of the network was tested in two ways. First, the network was tested using the same complete faces that were presented during training. Secondly, network performance was also tested by presenting either identity or expression in isolation. In both cases, we recorded the firing-rates from all neurons within the fourth (output) layer of the model.

### Information measures

The network's ability to recognise varying identities or expressions during testing is also assessed using information theory [37], [38]. However, the visual face stimuli used in the present study were constructed from a large number (40) of different expressions and identities in order to represent near continuous spaces of expression and identity. VisNet's information analysis measures were originally designed for a discrete number of visual stimuli, each of which was presented over a range of different transforms [37], [38]. Therefore, in order to apply these information measures to the current work, we needed to quantise the identity and expression spaces as follows.

In order to analyse how much information about facial identity was carried by neurons within the fourth (output) layer of the network, the 40 identities were quantised into five contiguous blocks of eight. During testing, we presented a central identity from within each of the five blocks, which was then combined with all 40 expressions. If a subpopulation of neurons in the fourth layer had learned to represent exclusively facial identity, then each neuron within that subpopulation should ideally respond selectively to only one of the five quantised identities. Furthermore, each neuron should respond to its favoured identity invariantly over all of the 40 expressions that can paired with that identity. In this case, such neurons will convey maximal information about facial identity.

In a separate analysis, the information measures were also used to assess how much information was carried by fourth layer neurons about facial expression. This was done by quantising and testing the expression space in a corresponding manner.

The single cell information measure has been used to show how much information is available from the response of a single cell about a stimulus that was presented over a range of different transforms [37], [38]. In the present study, the stimulus is taken to be one of the five quantised identities or five quantised expressions. When we are assessing the amount of information conveyed about identity, then the five quantised identities are treated as the five stimuli, and the 40 expressions are treated as transforms of those five identity stimuli. Conversely, when we are assessing the amount of information conveyed about expression, then the five quantised expressions are treated as the five stimuli, and the 40 identities are treated as transforms of those five expression stimuli.

The single cell information measure for each cell shows the maximum amount of information that the cell conveys about any one stimulus over all transforms. This is computed using the following formula with details provided elsewhere [37], [38]. The stimulus-specific information *I*(*s*, *R*) is the amount of information the set of responses R has about a specific stimulus s, and is given by
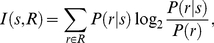
(6)where r is an individual response from the set of responses R.

The maximum amount of information that can be attained is 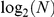 bits, where *N* is the number of stimuli. For the case of five stimuli, the maximum amount of information is 2.32 bits.
